# Neuroprotective Effects of Empagliflozin Against Methamphetamine-Induced Withdrawal Syndrome in Mice: Involvement of Bdnf, Tlr4, and JNK Signaling Pathways and Oxidative Stress Biomarkers

**DOI:** 10.5812/ijpr-172023

**Published:** 2026-07-07

**Authors:** Hamed Ghavimi, Mina Ebrahimi, Maede Hasanzadeh, Alireza Lashgari, Keivan Nedaei, Ali Kalantari Hesari, Mir-Jamal Hosseini

**Affiliations:** 1 Department of Pharmacology and Toxicology, School of Pharmacy, Zanjan University of Medical Sciences, Zanjan, Iran; 2 Department of Medical Biotechnology, Faculty of Medicine, Zanjan University of Medical Sciences, Zanjan, Iran; 3 Department of Pathobiology, Faculty of Veterinary Sciences, Bu-Ali Sina University, Hamedan, Iran

**Keywords:** Empagliflozin, Methamphetamine, Withdrawal Syndrome, Depression, Neuroprotection, Gene Expression

## Abstract

**Background:**

Methamphetamine withdrawal is associated with reduced dopaminergic neurotransmission, oxidative stress, neuroinflammation, and structural brain changes, which are linked to cognitive impairment, memory deficits, and psychological distress.

**Objectives:**

This study investigated whether empagliflozin, a sodium-glucose cotransporter 2 inhibitor, mitigates behavioral, molecular, and histopathological alterations induced by chronic methamphetamine (METH) exposure.

**Methods:**

Eighty male NMRI mice were allocated to 8 groups (n = 10). Methamphetamine dependence was induced by administering 2 mg/kg intraperitoneally, twice daily, for 14 days. During METH withdrawal, mice received empagliflozin (0.5, 1, 2, or 10 mg/kg, orally) or saline for 10 days. From days 25 to 28, the open-field test, forced swimming test, elevated plus maze, and novel object recognition test were conducted to assess depression-like behavior, anxiety-like behavior, and cognition. On day 28, the hippocampi were isolated and evaluated for oxidative and antioxidant biomarkers, nitric oxide (NO), and the expression of toll-like receptor 4 (Tlr4), brain-derived neurotrophic factor (Bdnf), and c-Jun N-terminal kinase (JNK). Histopathological studies of the hippocampus and prefrontal cortex were also performed using hematoxylin and eosin staining.

**Results:**

Methamphetamine withdrawal induced pronounced anxiety-like and depression-like behaviors, recognition memory impairment, redox imbalance, upregulation of Tlr4 and JNK, suppression of Bdnf, and inflammation. However, post-withdrawal empagliflozin ameliorated affective and cognitive deficits, restored glutathione and ferric-reducing antioxidant power, reduced malondialdehyde and protein carbonyl levels, normalized tissue NO levels, increased Bdnf expression, downregulated Tlr4 and JNK expression, and markedly reversed histopathological damage. These findings suggest that empagliflozin confers robust behavioral, antioxidant, anti-inflammatory, and neuroprotective effects during methamphetamine withdrawal.

**Conclusions:**

The results of this animal study support empagliflozin as a potential treatment for neuropsychiatric dysfunction associated with METH withdrawal. Further clinical studies are needed to confirm the effects of empagliflozin.

## 1. Background

Methamphetamine (METH) is one of the most widely abused and highly addictive stimulants. Recently, METH has emerged as a major drug of abuse worldwide. Although it can be produced through a simple synthetic pathway, current METH use surpasses that of cocaine and heroin (1). Increasing evidence suggests that the high abuse potential of METH arises from its ability to markedly elevate central nervous system (CNS) activity by increasing extracellular dopamine (2). Although investigations of METH have primarily focused on neurotransmitters and neuronal activity, accumulating evidence indicates that chronic METH exposure induces a broad range of harmful effects on the CNS, including neuroinflammation, oxidative stress, and excitotoxicity (2). The precise mechanisms of METH-induced neurotoxicity and neuroinflammation, particularly in dopaminergic nerve terminals, remain unclear. However, several fundamental studies have shown that METH induces damage through neuroinflammation and neurotoxicity associated with toll-like receptor 4 (Tlr4), microglial activation, oxidative stress, autophagy, and apoptosis (1). In addition, prolonged METH exposure increases reactive oxygen species, damages proteins, lipids, and DNA, suppresses endogenous cellular antioxidant defenses, and promotes neuronal loss, particularly in the hippocampus and amygdala (3). A substantial body of evidence suggests that prolonged use and abrupt cessation of METH trigger clinical problems, such as depression and anxiety, which are considered major symptoms of METH withdrawal (4-6).

Empagliflozin is a selective sodium-glucose cotransporter 2 (SGLT2) inhibitor prescribed for the management of obesity and type 2 diabetes, with established metabolic and pleiotropic benefits (7). Beyond glycemic control, empagliflozin exerts anti-inflammatory and antioxidant effects in cardiovascular, renal, neurological, and autoimmune systems (8). SGLT2 inhibitors can affect SGLT1/SGLT2 cotransporters, and previous preclinical studies have indicated that they are involved in neurogenesis, brain metabolism, neurotransmitter-release modulation, and cognition. Furthermore, findings from a meta-analysis indicated a positive effect of empagliflozin on cognitive function scores, particularly among populations with mild cognitive impairment or dementia. In addition, empagliflozin inhibits nuclear factor kappa B-dependent signaling, reduces interleukin 6 and tumor necrosis factor alpha, attenuates inflammation, improves mitochondrial function, and reduces reactive oxygen species production in the CNS (9-11).

## 2. Objectives

Because oxidative stress, neuroinflammation, and related gene-expression changes play major roles in METH withdrawal-induced depression and neurotoxicity, and because no targeted treatments currently address these mechanisms, there is a clear need to identify agents with combined antioxidant, anti-inflammatory, and neuroprotective properties. Accordingly, we investigated whether empagliflozin could mitigate METH withdrawal-associated molecular disturbances, including oxidative stress markers and the expression of Tlr4, JNK, and Bdnf, and whether these effects would translate into improvements in depressive-like and anxiety-like behaviors, as well as neuropathological alterations. Therefore, this study was designed to evaluate the protective effects of empagliflozin on behavioral, biochemical, genetic, and histopathological indices in an experimental model of METH withdrawal-associated depression.

## 3. Methods

### 3.1. Materials

Chemicals, including METH and fluoxetine, of the highest available analytical grade were purchased from Merck Co. (Germany). TRIzol reagent (Life Biolab) was used for RNA extraction, and diethyl pyrocarbonate-treated water was supplied by Sinaclon (Iran). First-strand complementary DNA synthesis was performed using the Sinaclon First Strand cDNA Synthesis Kit (RT5201; Sinaclon, Iran). Gene-specific primers for Tlr4, Bdnf, and JNK were purchased from Pishgam (Iran). Safe Stain loading dye, a DNA ladder, agarose, Tris base, ethylenediaminetetraacetic acid, loading buffer, and additional diethyl pyrocarbonate-treated water were supplied by Sinaclon (Iran). Quantitative reverse transcription polymerase chain reaction Master Mix and Mastermix Red for conventional polymerase chain reaction were purchased from Ampliqon (Denmark). Empagliflozin was obtained from Abidi Co. (Iran), and normal saline was provided by Iran Pharmaceutical and Injectable Products Company (Iran).

### 3.2. Animals and Housing Conditions

Male NMRI mice (22 - 25 g) were purchased from the Pasteur Institute in Tehran, Iran. The animals were housed in approved cages (4 animals per cage) under laboratory conditions, including an ambient temperature of 22 ± 2°C, relative humidity of 50% - 55%, and a 12:12-hour light/dark cycle, with ad libitum access to water and food throughout the experimental period. The mice were randomly divided into 8 experimental groups, with 10 mice in each group. In addition, 48 hours before the experiment, the animals were habituated to the testing environment. They were transferred to the experimental laboratory, weighed, and handled to facilitate adaptation to manipulation and minimize nonspecific stress responses. All tests were performed between 10:00 AM and 02:00 PM according to the National Institutes of Health Guide for the Care and Use of Laboratory Animals. All experimental procedures were conducted in accordance with the NIH Guide (NIH publication No. 80 - 23) for the Care and Use of Laboratory Animals. The protocols were approved by the institutional ethics committee on animal experimentation at Zanjan University of Medical Sciences (ethical code: IR.ZUMS.AEC.1402.042). All efforts were made to minimize the number of animals used and their suffering. All behavioral, biochemical, molecular, gene-expression, and histopathological investigations were performed by experimenters blinded to the identity of the experimental groups.

### 3.3. Methamphetamine-Induced Withdrawal Syndrome Model

In this study, mice were treated with intraperitoneal injections of METH (2 mg/kg) twice daily for 14 consecutive days, as described in our previously published papers (1, 5). The control groups were similarly injected intraperitoneally with normal saline twice daily to exclude solvent effects. Subsequently, the treated animals were kept in cages for 10 days without any METH or saline injections to induce withdrawal syndrome, which was confirmed through behavioral and molecular assessments.

### 3.4. Study Design

**Figure 1.**
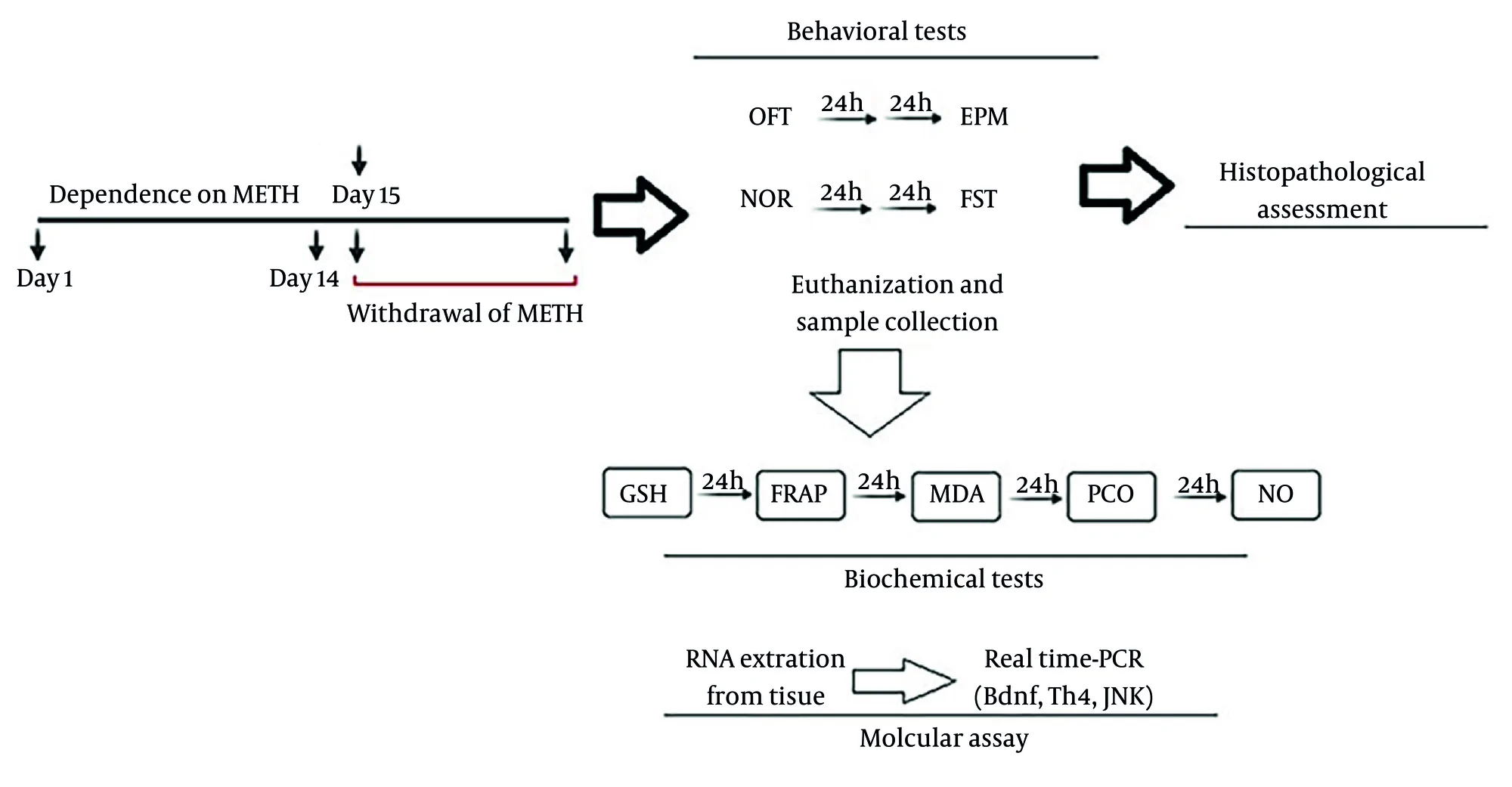
Schematic representation of the experimental design

A total of 80 mice were randomly divided into 8 groups (n = 10 in each group) and subjected to the following treatments: 1) the control group, in which mice received normal saline intraperitoneally twice daily for 14 consecutive days; 2) mice received METH (2 mg/kg intraperitoneally twice daily) for 14 consecutive days, with no further treatment during withdrawal (METH group); 3) normal mice did not receive METH and were treated with the highest dose of empagliflozin alone (10 mg/kg by oral gavage once daily for 10 days) to evaluate its direct effects under nonpathological conditions (empagliflozin group); groups 4, 5, 6, and 7 received daily intraperitoneal METH for 14 days and empagliflozin by oral gavage (0.5, 1, 2, and 10 mg/kg, respectively) during the 10 days of METH withdrawal; and 8) mice received METH (2 mg/kg intraperitoneally twice daily for 14 days) followed by fluoxetine (5 mg/kg by oral gavage once daily for 10 days) as a standard antidepressant comparator. The experimental design is summarized in Figure 1. From days 25 to 28, between 10:00 AM and 02:00 PM, standard behavioral approaches, including the open-field test (OFT), forced swimming test (FST), elevated plus maze (EPM), and novel object recognition (NOR) test, were used to evaluate depression-like behavior and anxiety levels in mice. Finally, the brains were rapidly harvested; the hemispheres were separated and rinsed with isotonic saline buffer. The hippocampus was used to assess oxidative stress biomarkers and mRNA expression of Bdnf, Tlr4, and JNK, and histopathological investigations were performed in the cerebral cortex and dentate gyrus.

### 3.5. Behavioral Studies

#### 3.5.1. Open-Field Test

The OFT was used to evaluate locomotor activity, exploratory behavior, and anxiety-like responses in mice. The apparatus consisted of a transparent Plexiglas box (50 × 50 × 50 cm), and the floor was virtually divided into equal squares. At the beginning of the test, each mouse was gently placed in the center of the box and allowed to move freely for 5 minutes. During this period, the number of squares crossed (horizontal activity), the number of rearings on the hind limbs, and the total time spent in the central area were recorded. Decreased locomotor activity and reduced time spent in the center were considered indices of increased anxiety or diminished exploratory motivation. After each trial, the floor and walls of the apparatus were cleaned with 70% ethanol to remove olfactory cues (5).

#### 3.5.2. Forced Swimming Test

The FST was used to assess depressive-like behavior in mice. This test was performed according to the standard protocol described in previous studies. Briefly, each animal was placed individually in a glass cylinder (14 × 40 cm) filled with water maintained at 25 ± 1°C. The total duration of the test was 6 minutes; however, only the last 4 minutes were analyzed for immobility time. Immobility was defined as the absence of active escape-directed behavior, with the animal making only minimal movements necessary to keep its head above the water surface. An increase in immobility duration was interpreted as behavioral despair and considered an index of depressive-like behavior. At the end of each session, the mice were gently dried with a towel and returned to a warmed cage to prevent additional stress. The water was replaced before testing the next animal to keep the experimental conditions constant (1).

#### 3.5.3. Elevated Plus Maze

The EPM is a well-established behavioral test for evaluating anxiety-related behavior in rodents. This test was performed according to the standard protocol reported in previous studies (12). The apparatus comprised 2 opposed open arms (50 × 10 × 2 cm) and 2 opposed closed arms (50 × 10 × 50 cm) connected by a central platform (10 × 10 cm) 60 cm above the floor. The tendency to remain in the closed arms rather than the open arms was representative of anxiety-like behavior, whereas the tendency to remain in the open arms rather than the closed arms reflected anxiolytic behavior. At the start of the test, each mouse was placed on the central platform facing one of the open arms and allowed to freely explore the maze for 5 minutes. An arm entry was counted when all 4 paws of the animal had entered that arm. The time spent in the open arms (OAT) and closed arms (CAT) was measured in seconds, and the percentage of time spent in the open arms was calculated using the following formula: OAT = OAT / (OAT + CAT) × 100. After each trial, the surface of the maze was cleaned and dried with 70% ethanol to eliminate olfactory traces (12).

#### 3.5.4. Novel Object Recognition Test

The NOR test was used to evaluate recognition memory and was performed in an opaque plastic box. The procedure consisted of 3 consecutive sessions: 1) habituation session: on day 1, each mouse was placed in the empty box for 10 minutes to habituate to the environment; 2) familiarization session: on day 2, 2 identical objects, similar in shape, color, and size, were placed in the left and right positions of the box, and each mouse was allowed 15 minutes to freely explore both objects; and 3) test session: on day 3, 1 of the familiar objects was replaced by a novel object. The novel object differed from the familiar one in shape and color while maintaining similar color contrast and accessibility for the animal. Each mouse was allowed to explore the familiar and novel objects for 3 minutes, and this session was video-recorded. The time spent exploring the familiar object and the novel object was measured in seconds, and the discrimination index (DI) was calculated as DI (%) = time spent exploring the novel object / time spent exploring both objects × 100. A ratio greater than 0.5 was considered to indicate intact memory and normal cognitive performance, whereas a ratio below 0.5 was interpreted as impaired cognitive function. After each test, the box was wiped with cotton soaked in 70% ethanol, and after approximately 1 minute, the next animal was introduced (13).

### 3.6. Biochemical and Molecular Assays

#### 3.6.1. Measurement of Glutathione Content in Hippocampal Tissue

Tissue glutathione (GSH) functions as a key intracellular antioxidant and the initial line of protection against excessive free-radical generation. Tissue GSH content was determined using 5,5′-dithiobis (2-nitrobenzoic acid) as the chromogenic reagent. Briefly, 0.1 g of hippocampal tissue was transferred to a homogenizer with 1 mL of ethylenediaminetetraacetic acid and 1.5 mL of trichloroacetic acid. Next, 1 mL of the supernatant was added to 2.5 mL of 0.4 mol/L phosphate buffer and 0.5 mL of 5,5′-dithiobis (2-nitrobenzoic acid) indicator (pH 8.9). The absorbance of the developed yellow chromophore was measured at 412 nm using a spectrophotometer, and GSH levels were expressed as µM/g tissue (13).

#### 3.6.2. Ferric-Reducing Antioxidant Power Assay

Total antioxidant capacity was evaluated using the ferric-reducing antioxidant power (FRAP) method, based on the reduction of Fe^3+^ to Fe^2+^, as originally described by Benzie and Strain. Hippocampal tissue (0.1 g) was dissected on ice and homogenized in 1 mL of 6% trichloroacetic acid. After centrifugation, 50 µL of the supernatant was added to 1.5 mL of freshly prepared FRAP reagent. The FRAP reagent was prepared by mixing 300 mM sodium acetate buffer (pH 3.6), 20 mM FeCl_3_·6H_2_O, and 10 mM 2,4,6-tri(2-pyridyl)-s-triazine dissolved in 40 mM HCl at a 10:1:1 ratio. The reaction mixture was incubated for 10 minutes in the dark, and the absorbance of the Fe^2+^-TPTZ complex was subsequently recorded at 593 nm using a UV-1601PC spectrophotometer. Ferric-reducing antioxidant power values were reported as mM/g tissue (1).

#### 3.6.3. Determination of Malondialdehyde Content in Hippocampal Tissue

Malondialdehyde (MDA), the main by-product of polyunsaturated fatty acid oxidation, is a known biomarker of lipid peroxidation principally induced by reactive oxygen species. Lipid peroxidation was assessed by quantifying MDA using the thiobarbituric acid reactive substances assay, with tetramethoxypropane serving as the calibration standard. Malondialdehyde reacted with thiobarbituric acid to produce a pink color, and its absorbance was measured spectrophotometrically at 532 nm. Malondialdehyde concentration was expressed as nM/g tissue (1).

#### 3.6.4. Determination of Protein Carbonyl Content in Hippocampal Tissue

Increased carbonylated protein-based reactions are another consequence of increased oxidative stress. To assess protein oxidation in hippocampal tissue, protein carbonyl groups were measured using the 2,4-dinitrophenylhydrazine derivatization method described by Oliver et al. The 2,4-dinitrophenylhydrazine-labeled products were quantified spectrophotometrically, and protein carbonyl content was calculated as nmol 2,4-dinitrophenylhydrazine incorporated per mg tissue using an extinction coefficient of 21 mM^-1^·cm^-1^ for aliphatic hydrazones (13).

#### 3.6.5. Determination of Nitric Oxide Concentration in Hippocampal Tissue

Nitric oxide concentration in hippocampal tissue was measured using a commercial NO assay kit (Zistfanteb, CIB Biotech, Iran). This assay is based on the Griess reaction, in which nitrate is first reduced to nitrite, and the resulting nitrite is subsequently quantified spectrophotometrically at 540 nm (13).

#### 3.6.6. Total RNA Extraction

Total RNA was extracted from rat hippocampal tissue (approximately 50 mg) using TRIzol reagent (Life Biolab, Iran) according to the manufacturer’s protocol. The quality and quantity of the extracted RNA were assessed by gel electrophoresis and spectrophotometry at 260:280 nm, respectively. For complementary DNA synthesis, 2 µL of extracted RNA was used as the template in a 20 µL reverse-transcription reaction prepared with the Sinaclon First Strand cDNA Synthesis Kit (RT520, Sinaclon, Iran), according to the supplier’s instructions (Table 1). The resulting complementary DNA was stored at -20°C until quantitative reverse transcription polymerase chain reaction analysis.

**Table 1.** Primer Sequences Used for Quantitative Reverse Transcription Polymerase Chain Reaction Assay

Name	Sequence (5′→3′)	GenBank
**Tlr4**	CCGCTCTGGCATCATCTTCA	NM_021297.3
	>TCCCACTCGAGGTAGGTGTT	
**Bdnf**	ATCCACTGAGCAAAGCCGAA	NM_007540.4
	CCTGGTGGAACATTGTGGCT	
**JNK**	TTACTGTGTCACGCCATGCT	NM_016700.5
	GAGCTTCTCTGTACTGGCGG	
**GAPDH**	ACTAACCCTGCGCTCCTG	NM_001256799.2
	CCCAATACGACCAAATCAGA	

#### 3.6.7. Gene Expression Assay by Quantitative Reverse Transcription Polymerase Chain Reaction

Quantitative real-time polymerase chain reaction was performed using SYBR Green Master Mix (Ampliqon, Denmark). Each 15 µL reaction contained 2 µL of complementary DNA (1 - 100 ng), 0.5 µL of forward primer, 0.5 µL of reverse primer, 7.5 µL of SYBR Green mix, and nuclease-free water to volume. The expression of Bdnf, Tlr4, JNK, and the reference gene GAPDH was evaluated using gene-specific primers (Table 1). The primer sequences were designed based on published data and checked for specificity using BLAST. Amplification was carried out with an initial denaturation at 95°C for 10 minutes, followed by 40 cycles of denaturation at 95°C for 30 seconds, annealing at 57°C for 30 seconds, and extension at 72°C for 30 seconds. Melting-curve analysis was performed at the end of the run to confirm that each primer pair yielded a single specific product. No-template controls, using water instead of complementary DNA, were included for both target and reference genes in each run. Relative mRNA expression levels of Bdnf, Tlr4, and JNK were calculated using the 2^-ΔΔCt^ method, with GAPDH used for normalization (1).

### 3.7. Histopathological Investigation

The cerebral cortex and dentate gyrus of the hippocampus were dissected and fixed in 10% formalin. Following serial dehydration in graded ethanol solutions, the tissues were stained with hematoxylin and eosin and examined under a light microscope (Olympus BX51) at 400× magnification. Five samples from each group were included in the histological evaluation, and images were captured using a camera mounted on the microscope. Histopathological changes were scored on a 5-point scale, in which 0 indicated no detectable change and 5 represented severe alteration.

### 3.8. Statistical Analysis

The sample size was calculated by power analysis using G*Power software with an alpha error of 0.05 and power (1 - β) of 0.8. The required total sample size per group was calculated as 7 - 8 for behavioral tests (n = 10 in each group) and 3 - 4 for molecular, biochemical, and histopathological studies. Results are presented as mean ± standard deviation. Statistical analyses were performed using SPSS software, version 17. Group differences were evaluated using 1-way analysis of variance followed by Tukey post hoc tests. Pairwise comparisons were conducted among all METH-dependent and nondependent groups. P < 0.05 was considered statistically significant.

## 4. Results

### 4.1. Behavioral Experiments

4.1.1. Effects of Empagliflozin on Depressive-Like Behaviors Following Methamphetamine Withdrawal Syndrome in Mice: Open-Field Test

**Figure 2.**
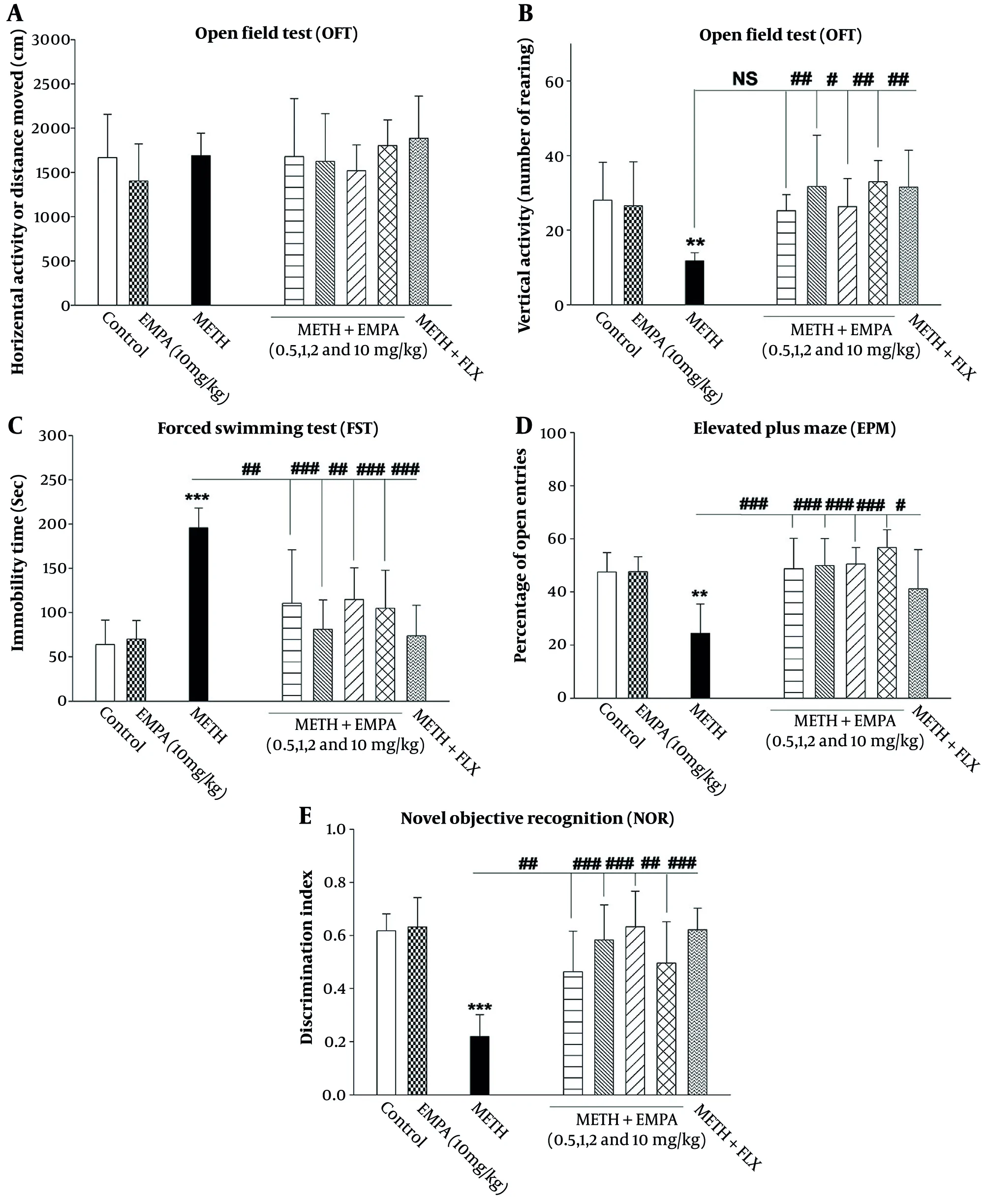
Effects of methamphetamine and empagliflozin on (A) horizontal activity and (B) vertical activity in the open-field test; (C) immobility time in the forced swimming test; (D) anxiety-like behavior in the elevated plus maze; and (E) cognitive performance in the novel object recognition test. Data are expressed as mean ± standard deviation (n = 10). One-way analysis of variance followed by Tukey post hoc tests was used to determine statistical significance. Symbols denote: ** P < 0.01 and *** P < 0.001 vs the control group; # P < 0.05, ## P < 0.01, and ### P < 0.001 vs the METH group.

The OFT data revealed no significant effect of any treatment on horizontal activity (distance traveled). As presented in Figure 2A, chronic METH administration alone did not alter horizontal locomotor activity compared with the control group (F(7, 55) = 0.80; P = 0.591). Empagliflozin alone (10 mg/kg) likewise did not induce a significant change in this parameter (P > 0.05). In addition, combined treatment with METH and empagliflozin at 0.5, 1, 2, or 10 mg/kg, as well as fluoxetine as the positive control, did not show any significant difference in horizontal activity relative to the METH group (P > 0.05). These findings indicate that METH, empagliflozin, and fluoxetine did not exert a major effect on overall locomotor activity in male NMRI mice. Thus, the current METH regimen did not cause locomotor impairment at the time of behavioral analysis.

In contrast, vertical activity in the OFT differed significantly among groups. As illustrated in Figure 2B, METH alone produced a marked reduction in vertical exploration (rearing) compared with the control group (F(7, 55) = 3.952; P = 0.002). Empagliflozin inhibited the effect of METH at all doses and increased the number of rearings in the experimental animals. Moreover, there were no statistically significant differences in the number of rearings between any empagliflozin-treated group and the control animals (P = 0.999; P = 0.993; P = 1.000; P = 0.965; or P > 0.05). Nonetheless, empagliflozin at doses of 1, 2, and 10 mg/kg significantly improved the number of rearings compared with the group that received only METH (P < 0.01, P < 0.05, and P < 0.01, respectively). Fluoxetine showed a similar pattern, with a significant increase in the number of rearings relative to the METH group (Figure 2B; P < 0.01). These data suggest that empagliflozin effectively attenuates the anxiety-like consequences of METH withdrawal by restoring exploratory behavior in male NMRI mice.

4.1.2. Effects of Empagliflozin on Immobility Time During the Forced Swimming Test in the Experimental Groups Following Methamphetamine Withdrawal Syndrome

After 10 days of withdrawal induction following METH exposure, swimming time in mice treated with intraperitoneal METH was dramatically decreased in the FST compared with the control group (F(7, 55) = 9.471; P < 0.001; Figure 2C). Oral administration of empagliflozin at all doses used in this study following METH withdrawal significantly reduced immobility time in the FST compared with the METH-only group (P < 0.001 for all; Figure 2C), indicating an antidepressant-like effect of empagliflozin in this experimental animal model. Similar results were observed for immobility time in the FST when fluoxetine was administered orally as a positive control compared with the METH-treated group (P < 0.001).

4.1.3. Results of Anxiety-Like Behavior in the Elevated Plus Maze in Control and Treatment Groups Following Methamphetamine Withdrawal Syndrome

The EPM was conducted to further assess anxiety-like behavior in this study. As shown in Figure 2D, mice in the control group spent more time in the open arms of the EPM and less time in the closed arms than mice treated with only 2 mg/kg METH (P < 0.01), reflecting increased anxiety-like behavior in METH-dependent mice. The results indicated that post-METH withdrawal administration of empagliflozin at doses of 0.5, 1, 2, and 10 mg/kg led to a significant increase in time spent in the open arms of the EPM compared with the METH-only group (F(7, 55) = 7.052; P < 0.01 for 0.5 mg/kg empagliflozin and P < 0.001 for 1, 2, and 10 mg/kg empagliflozin; Figure 2D). Fluoxetine also significantly increased the time spent in the open arms relative to the METH-only group (P < 0.05; Figure 2D).

4.1.4. Effects of Empagliflozin on Recognition Memory in the Novel Object Recognition Test in Different Experimental Groups Following Methamphetamine Withdrawal Syndrome

The NOR test was used to examine recognition memory and cognitive performance. One-way analysis of variance demonstrated a significant effect of treatment on the discrimination index. As presented in Figure 2E, METH-treated mice exhibited a pronounced reduction in the discrimination index compared with the control group (F(7, 55) = 10.137; P < 0.001), indicating impaired novel object recognition memory. Moreover, treatment with empagliflozin at 0.5, 1, 2, and 10 mg/kg significantly increased the discrimination index relative to the METH group (P < 0.01 for 0.5 and 10 mg/kg empagliflozin and P < 0.001 for 1 and 2 mg/kg empagliflozin). Fluoxetine (5 mg/kg) likewise produced a significant increase in the discrimination index compared with the METH group (P < 0.001).

### 4.2. Biochemical Assays

4.2.1. Effect of Empagliflozin on Methamphetamine Withdrawal-Induced Alterations in Oxidative Stress Markers in the Mouse Hippocampus

As illustrated in Table 2, mice in the METH-only group demonstrated significant elevations in lipid peroxidation, as indicated by increased MDA levels (F(7, 23) = 49.586; P < 0.001), and in protein carbonyl content, a biomarker of protein oxidation, compared with control mice (F(7, 23) = 38.040; P < 0.001). The findings also showed that FRAP, a biomarker of total antioxidant capacity, and GSH, a nonenzymatic antioxidant biomarker, were considerably decreased in METH-only mice, indicating a significant decrease in total antioxidant power in hippocampal tissue compared with the control group (F(7, 23) = 5.915; P = 0.002 and F(7, 23) = 29.488; P < 0.001, respectively; Table 2). Conversely, statistical analysis showed that various doses of empagliflozin (0.5, 1, 2, and 10 mg/kg) reduced the METH-induced increases in MDA and protein carbonyl levels and inhibited the METH-induced decreases in GSH and FRAP compared with the METH-only group (P < 0.001). Interestingly, the levels of MDA, protein carbonyl, GSH, and FRAP in METH-dependent mice treated with fluoxetine (5 mg/kg) and various doses of empagliflozin did not differ statistically (Table 2).

**Table 2.** Effects of Various Doses of Empagliflozin on Alterations in Oxidative Stress Biomarkers in Mouse Hippocampal Tissue Treated With Methamphetamine (2 mg/kg) ^a, b^

Groups	MDA (nM/g Tissue)	FRAP (mM/g Tissue)	GSH (µM/g Tissue)	Protein Carbonyl (µM/g Tissue)
**Control**	118.4 ± 7.9	430.7 ± 29.3	217.3 ± 15.5	1.02 ± 0.004
**EMPA (10 mg/kg)**	117.9 ± 8.1	442.4 ± 28.1	219.2 ± 5.2	0.86 ± 0.08
**METH (2 mg/kg)**	203 ± 5.7***	192.7 ± 33***	72.2 ± 3.4***	2.09 ± 0.11***
**METH + EMPA (0.5 mg/kg)**	164.2 ± 10.4***###	456.3 ± 14.17##	117.4 ± 5.8***###	1.16 ± 0.08###
**METH + EMPA (1 mg/kg)**	147.5 ± 10**###	412.6 ± 67.6##	192.6 ± 34.3###	1.22 ± 0.07###
**METH + EMPA (2 mg/kg)**	139.4 ± 8.9###	412.2 ± 65.2##	216.5 ± 25.2###	1.17 ± 0.12###
**METH + EMPA (10 mg/kg)**	112 ± 4.6###	386.2 ± 30.5#	180.8 ± 8.6###	1.36 ± 0.06*###
**METH + FLX**	117.3 ± 1.7###	415.1 ± 9.48##	209.8 ± 14.9###	1.01 ± 0.21###

^a^ Values are expressed as the mean±SD (n=4). One-way ANOVA followed by Tukey’s post hoc tests was employed to determine statistical significance.

^b^* P < 0.05, ** P < 0.01, *** P < 0.001 vs. control group; # P < 0.05, ## P < 0.01, ### P < 0.001 vs. METH group.

#### 4.2.2. Effect of Empagliflozin on Methamphetamine Withdrawal-Induced Alterations in Hippocampal Nitric Oxide Levels

**Figure 3.**
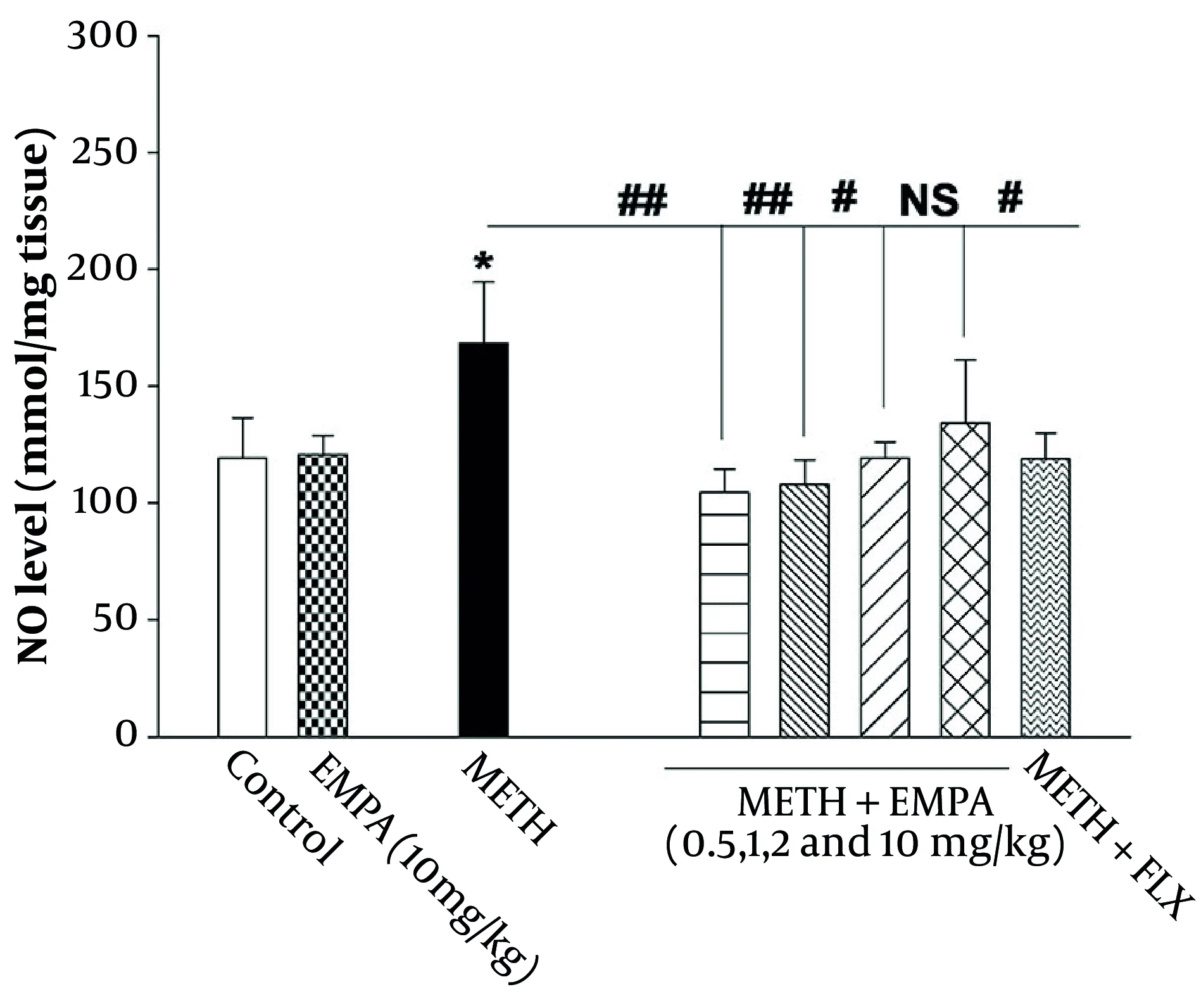
Effects of methamphetamine and empagliflozin treatment on nitric oxide levels in the hippocampus of mice. Data are expressed as mean ± standard deviation (n = 4). One-way analysis of variance followed by Tukey post hoc tests was used to determine statistical significance. Symbols denote: * P < 0.05; # P < 0.05 and ## P < 0.01.

Statistical analysis indicated that the treatment regimen markedly affected NO levels in the mouse hippocampus. As shown in Figure 3, METH-only mice showed a significant increase in NO concentration compared with the control group (F(7, 23) = 4.439; P = 0.006), suggesting hyperactivation of nitrergic signaling during the withdrawal period. Administration of empagliflozin alone (10 mg/kg) to non-METH-exposed mice did not alter hippocampal NO levels compared with control animals (P = 1.000; P > 0.05), indicating that empagliflozin does not affect basal NO homeostasis under physiological conditions.

Compared with the METH group, post-withdrawal treatment with empagliflozin at 0.5, 1, and 2 mg/kg significantly decreased hippocampal NO levels (P < 0.01 for 0.5 and 1 mg/kg empagliflozin and P < 0.05 for 2 mg/kg empagliflozin vs METH-only mice), partially restoring them toward control values. In contrast, the highest dose of empagliflozin (10 mg/kg) did not significantly modify NO levels compared with METH-treated mice (P = 0.243 or P > 0.05), despite exhibiting numerically similar values to the control group, suggesting a loss of efficacy at this upper dose. Fluoxetine (5 mg/kg), used as a positive control, significantly attenuated hippocampal NO concentration in METH-exposed mice (P < 0.05 vs METH-only mice) and normalized values to those of control animals (P = 1.000 or P > 0.05 vs control). Collectively, these findings suggest that low-to-intermediate doses of empagliflozin can attenuate METH-induced alterations in hippocampal NO levels, exhibiting a normalizing profile comparable to that of fluoxetine.

### 4.3. Gene Expression

4.3.1. Effects of Empagliflozin on Methamphetamine Withdrawal-Induced Alterations in Tlr4 and JNK mRNA Expression in the Mouse Hippocampus

**Figure 4.**
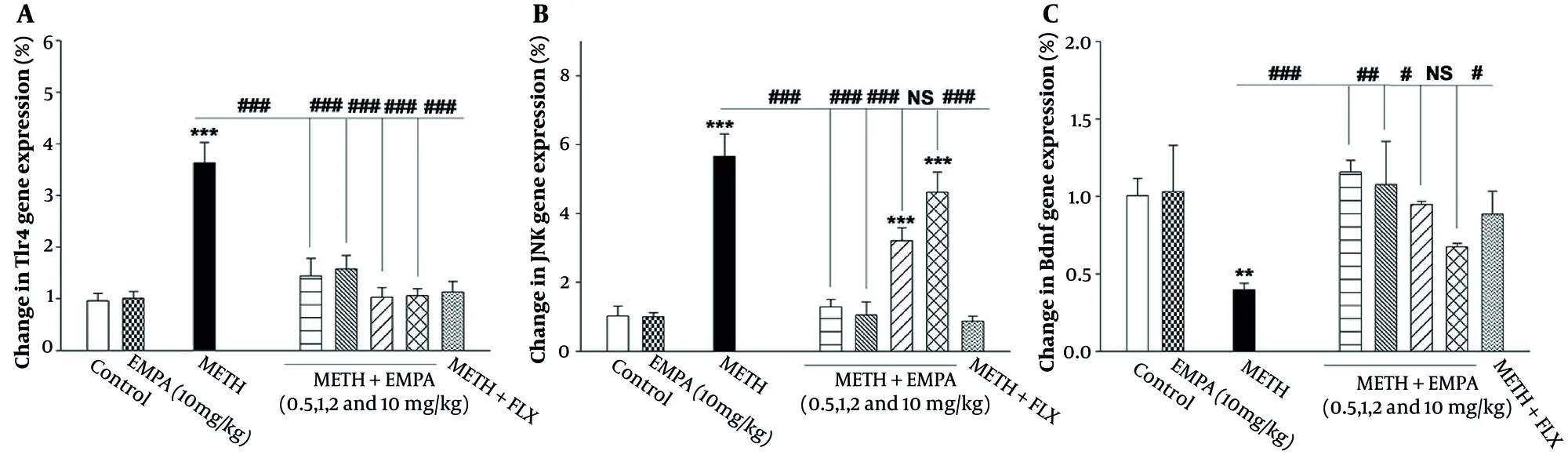
Effects of methamphetamine and empagliflozin treatment on (A) Tlr4, (B) JNK, and (C) Bdnf gene expression in the hippocampus. Data are expressed as mean ± standard deviation (n = 3). One-way analysis of variance followed by Tukey post hoc tests was used to determine statistical significance. Symbols denote: ** P < 0.01 and *** P < 0.001 vs the control group; # P < 0.05, ## P < 0.01, and ### P < 0.001 vs the METH group.

One-way analysis of variance showed significant effects of METH (2 mg/kg) withdrawal induction on Tlr4 (F(7, 23) = 41.418; P < 0.001) and Bdnf (F(7, 23) = 71.408; P < 0.001) gene expression. Tlr4 expression in the METH-only group was markedly increased compared with the control group, which was treated only with saline (P < 0.001; Figure 4A), indicating robust induction of this inflammatory marker following METH exposure. Administration of empagliflozin at 0.5, 1, 2, and 10 mg/kg significantly reduced Tlr4 gene expression compared with the METH group (P < 0.001 for all doses of empagliflozin; Figure 4A), such that values in the treated groups approached those of controls. Fluoxetine treatment produced a similar pattern, with Tlr4 expression lower than that in the METH group. These findings are consistent with a prominent inhibitory effect of empagliflozin on Tlr4-dependent inflammatory pathways and mitigation of METH-induced oxidative stress.

As shown in Figure 4B, JNK expression in the METH-only group was markedly higher than that in the control group (F(7, 23) = 71.408; P < 0.001), reflecting strong activation of stress- and inflammation-related signaling pathways in the mouse hippocampus after METH exposure. In contrast, JNK expression in the control group and in animals receiving empagliflozin 10 mg/kg alone remained low and close to baseline levels. Treatment with empagliflozin at 0.5, 1, and 2 mg/kg during METH withdrawal significantly decreased JNK expression compared with the METH-only group (P < 0.001 for all), indicating robust suppression of METH-induced inflammatory and stress signaling. As illustrated in Figure 4B, administration of empagliflozin at 10 mg/kg during METH withdrawal did not reverse the METH-induced effects on hippocampal JNK gene expression. Fluoxetine treatment significantly lowered JNK expression relative to the METH group (P < 0.001), similar to the inhibitory profile of the lower empagliflozin doses, confirming its role as a modulator of inflammation-related signaling.

4.3.2. Effects of Empagliflozin on Methamphetamine Withdrawal-Induced Alterations in Bdnf mRNA Expression in the Mouse Hippocampus

Twice-daily intraperitoneal administration of METH (2 mg/kg) markedly reduced Bdnf gene expression in the rat hippocampus compared with the control group, suggesting impaired neurogenesis and synaptic function (F(7, 23) = 7.096; P < 0.001; Figure 4C). Conversely, the doses of empagliflozin used in this study (0.5, 1, and 2 mg/kg) during METH withdrawal significantly altered hippocampal mRNA expression of Bdnf in METH-treated animals compared with the METH-only group (P < 0.001, P < 0.01, and P < 0.05, respectively; Figure 4C). The standard antidepressant fluoxetine also reduced the METH-induced decrease in hippocampal Bdnf gene expression compared with the METH-only group; however, its effect was less than that of empagliflozin at 0.5 and 1 mg/kg (Figure 4C). Overall, these data support a neurotrophic and neuroprotective role for empagliflozin against METH withdrawal-related damage.

### 4.4. Effects of Treatments on Histopathological Alterations in the Cerebral Cortex and Dentate Gyrus

**Figure 5.**
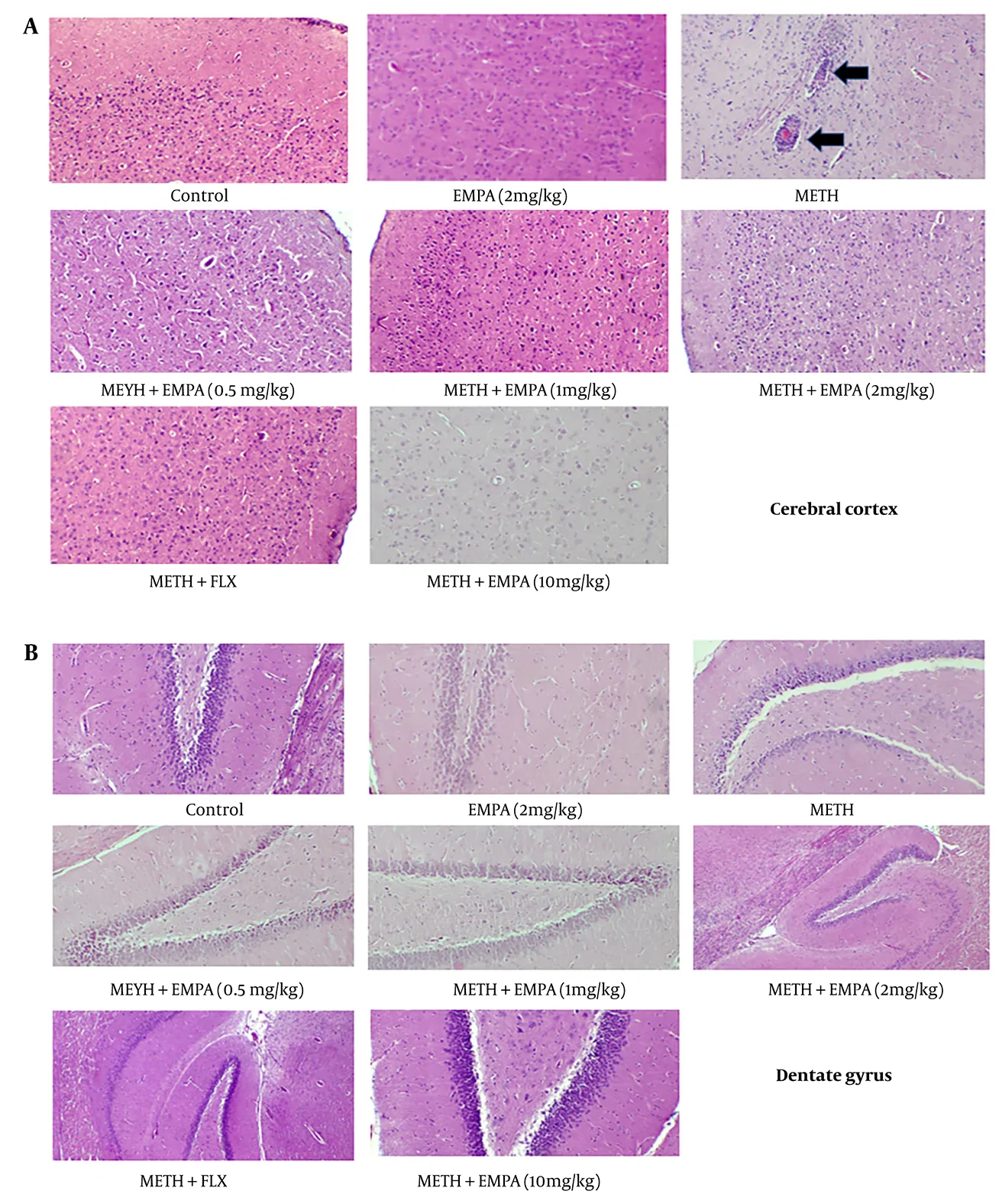
Histopathological alterations in the cerebral cortex and dentate gyrus following METH withdrawal and post-withdrawal treatments. (A) *Representative hematoxylin* and eosin-stained sections of the cerebral cortex from the control, empagliflozin (EMPA) 2 mg/kg, METH, METH + EMPA 0.5 mg/kg, METH + EMPA 1 mg/kg, METH + EMPA 2 mg/kg, and METH + fluoxetine 5 mg/kg groups. Mild inflammatory cell accumulation is evident in the METH group, whereas cortical architecture appears normal in the control, EMPA-alone, all METH + EMPA, and METH + fluoxetine groups. (B) *Representative hematoxylin* and eosin-stained sections of the dentate gyrus from the same experimental groups. Marked basophilic neuronal necrosis is observed in the METH group, which is reduced in a dose-dependent manner in the METH + EMPA 0.5 and 1 mg/kg groups and completely prevented in the METH + EMPA 2 mg/kg and METH + fluoxetine groups. Histopathological changes in both regions were graded on a semiquantitative scale from 0 (no change) to 5 (severe change), as summarized in Table 3. Magnification: ×400.

As illustrated in Figure 5A and Table 3, chronic METH administration produced evident inflammatory changes in the cerebral cortex. Mice in the METH group showed mild inflammatory cell accumulation (score 2), whereas the cerebral cortex of control animals and those receiving empagliflozin alone (2 mg/kg) exhibited normal histological architecture with no detectable inflammatory infiltrates (score 0). Post-withdrawal treatment with empagliflozin at all doses tested (0.5, 1, and 2 mg/kg), as well as fluoxetine (5 mg/kg), completely prevented METH-related cortical inflammation, with inflammatory scores reduced to 0, comparable to the control and empagliflozin-alone groups (Figure 5A and B and Table 3).

**Table 3.** Grading of Histopathological Changes in the Cerebral Cortex and Dentate Gyrus of Mice

Groups	Cerebral cortex (Inflammatory cell accumulation)	Dentate gyrus (Basophilic necrosis)
**Control**	0	0
**EMPA (10 mg/kg)**	0	0
**METH (2 mg/kg)**	2	3
**METH + EMPA (0.5 mg/kg)**	0	2
**METH + EMPA (1 mg/kg)**	0	1
**METH + EMPA (2 mg/kg)**	0	0
**METH + EMPA (10 mg/kg)**	0	0
**METH + FLX**	0	0

Regarding the dentate gyrus, Figure 5B and Table 3 show that METH exposure induced pronounced basophilic neuronal necrosis (score 3), whereas control and empagliflozin-alone mice displayed preserved cytoarchitecture without necrotic changes (score 0). Post-withdrawal administration of empagliflozin attenuated these degenerative alterations in an apparently dose-dependent manner: basophilic necrosis decreased from moderate (score 2) in the METH + empagliflozin 0.5 mg/kg group to mild (score 1) in the METH + empagliflozin 1 mg/kg group and was fully normalized (score 0) in the METH + empagliflozin 2 mg/kg group. Similarly, the dentate gyrus structure in METH + fluoxetine-treated mice was indistinguishable from that of controls (score 0). Overall, these findings indicate that empagliflozin, particularly at 2 mg/kg, effectively preserves cortical and hippocampal histological integrity during METH withdrawal, providing protection comparable to fluoxetine.

Scoring was performed from grade 0 (no change) to 5 (severe change).

## 5. Discussion

The present study demonstrates that METH withdrawal induces a robust constellation of behavioral, molecular, and structural abnormalities and that empagliflozin, particularly at 1 mg/kg, effectively mitigates these dysfunctions. Consistent with the known neurochemical profile of METH, which releases norepinephrine at approximately twice the level of dopamine and nearly 60-fold more than serotonin, the withdrawal state produced marked anxiety-like and depression-like behaviors, including reduced OFT rearing, increased immobility in the FST, diminished open-arm exploration in the EPM, and impaired recognition memory in the NOR task (14, 15). These effects align with the established roles of serotonin in mood, higher cognitive processes, and irritability, and norepinephrine in cognition and memory consolidation (16), supporting the expectation that disruption of monoaminergic tone during chronic METH exposure and withdrawal contributes to affective and cognitive decline. Moreover, the behavioral impairments observed here accord with primate studies showing long-lasting METH-induced neuronal injury (17) and profound dopaminergic loss following high-dose exposure (18), reinforcing the notion that withdrawal precipitates sustained neurobiological stress.

At the molecular level, METH withdrawal shifted the oxidative environment toward significant redox imbalance, as evidenced by depletion of GSH and FRAP and concomitant increases in MDA and protein carbonyl content. These findings correspond with the literature describing METH-associated neurotoxicity, oxidative stress, and the emergence of mood and cognitive vulnerabilities (19, 25). The observed upregulation of Tlr4 and JNK further reflects activation of oxidative stress-responsive inflammatory pathways, consistent with evidence that Tlr4 is upregulated through nuclear factor kappa B and mitogen-activated protein kinase signaling under oxidative conditions, thereby amplifying inflammatory cytokine production, blood-brain barrier disruption, and neurodegeneration (20-22). Similarly, the elevation of JNK is consistent with its role as a key mitogen-activated protein kinase family member activated by oxidative stress to promote mitochondrial permeabilization, cytochrome c release, and phosphorylation of Bcl-2 and Bax, shifting the balance toward apoptosis and tissue injury (23, 24). These molecular disturbances appear to underlie the observed histopathological changes characterized by cortical inflammatory cell infiltration and dentate gyrus neuronal necrosis.

Empagliflozin treatment during the withdrawal phase markedly improved this multidimensional pathological profile. The most pronounced benefits were observed at 1 - 2 mg/kg, where behavioral abnormalities were reversed, antioxidant markers were normalized, lipid and protein oxidation decreased, Bdnf expression was dramatically upregulated, and Tlr4 and JNK expression were significantly downregulated. These effects align closely with recent data indicating that SGLT2 inhibitors possess antidepressant and neuroprotective potential. Shimizu et al. (26) reported a 55-year-old woman with long-standing depression and type 2 diabetes whose glycemic variability and depressive symptoms improved after initiation of the SGLT2 inhibitor ipragliflozin L-proline, suggesting that SGLT2 inhibitors may exert antidepressant effects in patients with diabetes (26). Preclinical studies have also shown that empagliflozin reduces amyloid and tau pathology while increasing cortical Bdnf expression in transgenic diabetic mice. Other investigations have shown that empagliflozin reduced cortical amyloid burden, tau pathology, and brain atrophy and improved cognition in mice, possibly through increased Bdnf. Sodium-glucose cotransporter 2 inhibition may modulate atherosclerotic risk and stroke-related pathways through mechanisms involving proinflammatory mediators, thereby preserving the neurovascular unit and preventing maladaptive remodeling in diabetic brains (27). The current restoration of Bdnf expression by low-to-intermediate doses of empagliflozin parallels these findings and is particularly relevant given the central role of Bdnf in neuroplasticity, neurogenesis, synaptic function, cognition, and mood regulation (28-31).

The present histopathological findings further support the neuroprotective potential of empagliflozin. At effective doses, empagliflozin prevented cortical infiltration and reversed dentate gyrus necrosis, consistent with previous demonstrations that SGLT2 inhibition attenuates neuronal apoptosis, inflammation, and oxidative damage. Although recent pharmacokinetic analyses did not detect empagliflozin in brain tissue using conventional high-performance liquid chromatography methods (32), complementary docking data suggest that empagliflozin can modulate equilibrative nucleoside transporter 1 and thereby influence extracellular adenosine levels, a mechanism that may contribute to neuroprotection even in the absence of measurable CNS penetration. This interpretation is further supported by interim data from an ongoing clinical trial showing that empagliflozin reduces depressive symptoms in individuals with major depressive disorder (20), aligning with the behavioral improvements observed in this withdrawal model.

One notable finding is that the highest dose of empagliflozin (10 mg/kg) provided less consistent benefit across several biochemical and gene-expression outcomes. Given that SGLT2 inhibitors can strongly modulate multiple redox pathways, excessive stimulation may induce compensatory metabolic or inflammatory responses, thereby blunting therapeutic effects. This interpretation is supported by partial normalization of oxidative markers, attenuated Bdnf restoration, and less robust NO recovery at this dose, collectively suggesting a narrow dose range in which metabolic, antioxidant, and neurotrophic pathways are optimally regulated.

### 5.1. Study Limitations

Although these conclusions are convincing, several limitations should be considered. This study used only male NMRI mice, limiting generalizability across sexes and genetic backgrounds. Gene-expression analyses were confined to selected oxidative and inflammatory pathways, and protein-level confirmation was not performed. Detailed redox and mitochondrial metrics, as well as broader cytokine profiles, were not assessed, and only a single antidepressant comparator was included. Future work would benefit from expanded molecular profiling, time-course analyses, investigation of mitochondrial dynamics, inclusion of additional doses around the 1 - 2 mg/kg range, and evaluation of sex differences and alternative antidepressants. Integration of immunohistochemical and electrophysiological approaches may also clarify cell-type-specific and synaptic contributions to the neuroprotective actions of empagliflozin.

This study has the following potential limitations: 1) Estrus, especially the time of ovulation, involves complex behavioral changes, and hormonal changes during different estrous cycles may alter behavioral and molecular responses in animals. We did not evaluate the effects of METH withdrawal syndrome in female mice or the possible therapeutic effect of empagliflozin in this condition; and 2) western blotting validates gene-expression studies. The lack of protein-level confirmation by western blotting was the second limitation of this study. The authors could not conduct western blotting because of financial limitations.

### 5.2. Conclusions

In summary, our findings demonstrate that METH withdrawal induces a layered pattern of oxidative, inflammatory, metabolic, neurotrophic, behavioral, and structural dysfunction in the mouse hippocampus. Empagliflozin, particularly at low doses, counteracts these disturbances through a coordinated mechanism involving reinforcement of antioxidant capacity, including normalization of GSH and FRAP levels and reduction of MDA and protein carbonyl levels; attenuation of oxidative and inflammatory signaling, including downregulation of Tlr4 and JNK; and restoration of neurotrophic support through Bdnf. These molecular improvements translate into behavioral recovery and preservation of cortical and hippocampal integrity, underscoring the functional relevance of the biochemical effects. When integrated with expanding preclinical and clinical evidence supporting the neuroprotective and antidepressant properties of SGLT2 inhibitors (26, 27, 33, 34), our results position empagliflozin as a promising therapeutic candidate for mitigating the neurobiological consequences of METH withdrawal and potentially other mood and cognitive disorders driven by oxidative and inflammatory stress.

## Data Availability

The dataset presented in the study is available on request from the corresponding author during submission or after publication.
